# A General Life History Theory for Effects of Caloric Restriction on Health Maintenance

**DOI:** 10.1186/1752-0509-5-78

**Published:** 2011-05-19

**Authors:** Chen Hou, Kendra Bolt, Aviv Bergman

**Affiliations:** 1 Department of Systems and Computational Biology, Albert Einstein College of Medicine, Bronx, NY 10461, USA; 2 Key Laboratory of Agricultural Engineering in Structure and Environment, China Agriculture University, Beijing, China

## Abstract

**Background:**

Caloric restriction (CR) has been shown to keep organisms in a relatively youthful and healthy state compared to ad libitum fed counterparts, as well as to extend the lifespan of a diverse set of organisms. Several attempts have been made to understand the underlying mechanisms from the viewpoint of energy tradeoffs in organisms' life histories. However, most models are based on assumptions which are difficult to justify, or are endowed with free-adjusting parameters whose biological relevancy is unclear.

**Results:**

In this paper, we derive a general quantitative, predictive model based on physiological data for endotherms. We test the hypothesis that an animal's state of health is correlated with biological mechanisms responsible for the maintenance of that animal's functional integrities. Such mechanisms require energy. By suppressing animals' caloric energy supply and biomass synthesis, CR alters animals' energy allocation strategies and channels additional energy to those maintenance mechanisms, therefore enhancing their performance. Our model corroborates the observation that CR's effects on health maintenance are positively correlated with the degree and duration of CR. Furthermore, our model shows that CR's effects on health maintenance are negatively correlated to the temperature drop observed in endothermic animals, and is positively correlated to animals' body masses. These predictions can be tested by further experimental research.

**Conclusion:**

Our model reveals how animals will alter their energy budget when food availability is low, and offers better understanding of the tradeoffs between growth and somatic maintenance; therefore shedding new light on aging research from an energetic viewpoint.

## Background

Caloric restriction (CR), designed to induce "undernutrition without malnutrition" [1] usually reduces food intake to 20%-50% less than ad libitum levels, and has been the single most important environmental intervention shown to extend the lifespan in invertebrates and vertebrates (e.g. see [1-3]). In addition to extending lifespan, CR has been shown to prevent age-associated diseases and keep organisms in a relatively youthful and healthy state compared to ad libitum fed counterparts. These observations suggest that the somatic maintenance functions (e.g., cellular error-checking and damage repair) may be up-regulated in animals under CR conditions [2,4-9]. Several life history models have been proposed, using energy allocation strategies to explain how CR enhances the maintenance functions [10-14]. Most of these models are built on Disposable Soma Hypothesis [15-18], which suggests that an animal must budget its energy priorities amongst somatic maintenance, growth, activity, and reproduction. Since energy supply is limited and somatic maintenance (repair of molecular and cellular damage) is energetically costly, the organism must compromise. Therefore, maintenance cannot be perfect and damage is accumulated, which contributes to aging. The Disposable Soma Hypothesis further assumes that when additional energy is channeled to biological pathways of maintenance, an organism will have a better health status and will live longer. By suppressing other life history traits such as reproductive effort, CR channels energy to maintenance. These models qualitatively showed how CR enhances maintenance. However, there exist some shortcomings in these models. First, most models presume the energy tradeoffs between maintenance and other life history traits, instead of their being an outcome of the model. Second, it is difficult to justify such tradeoffs using physiological data [19]. Third, the spectrum of the empirical observations on CR that can be explained by some of these models is limited [19]. Finally, most of these models heavily depend on free-adjusting parameters, which have no biological meaning, or again, are difficult to link to real physiological data.

In this paper, we propose a quantitative model based on measurable physiological parameters and the concept of energy tradeoffs between biomass synthesis and maintenance for endothermic animals. Previous studies have shown that CR alters biomass synthesis in animals [e.g. see [20]]. This results in either retarded growth if CR conditions begin early in life, or loss of body mass if CR occurs later in life. The model proposed here handles both cases. To illustrate the basic idea of the model, we are going to discuss the case in which CR starts early in life and retards growth. The body mass reduction due to late-starting CR in adulthood can be treated (and will be referred to) as negative growth.

**Figure 1:**
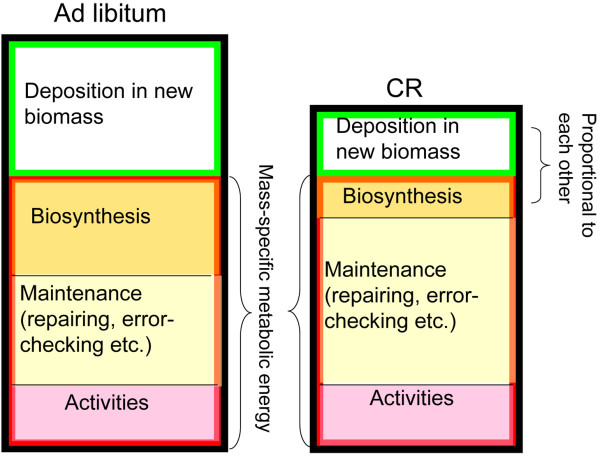
**Simplified illustration of energy partitioning in ad libitum fed and CR animals**. Box with black frame: rate of energy from food intake; box with red frame: metabolic rate; box with green frame: rate of energy deposition into new biomass. All quantities are mass-specific.

During growth, the energy assimilated from food is partitioned between the metabolic energy and energy deposited as new biomass (biomass gain) (Figure 1) [21]. The metabolic energy is further partitioned between energy for synthesizing new biomass; maintaining existing biomass, including damage repairing, error-checking et cetera; and normal activities, including locomotion, feeding, et cetera [21,22]. It is important to recognize the relationship between the energy deposited into new biomass and energy needed for the synthesis of new biomass. The former is the accumulated energy content of new biomass, while the latter is the metabolic work required to synthesize the new biomass, which corresponds to the indirect (organizational) work of growth [23], and is completely dissipated as heat, not conserved in fixed biomass. These two compartments are linearly proportional to each other [21,24].

When animals are under CR, the total mass-specific energy assimilated from food decreases (Figure 1, boxes with black frame). However, two mass-specific energy consumption rates remain roughly unchanged. One is the metabolic rate and the other is the level of activity. Numerous experiments on CR in mammals, including rodents, ewes, dogs, and primates, have shown that mass-specific metabolic rates of CR animals, expressed in per-gram of lean mass or per-gram of body mass to 3/4 power, *m*^3/4^, (metabolic mass), either decrease slightly [25-27] or remain the same as in ad libitum fed counterparts [28-36]. Under severe CR (50% or 60%), the mass-specific metabolic rate may drop, in some cases, by up to 15% [25,31,37-39]. One such study showed that the mass-specific metabolic rate may be even higher in CR animals [33]. Regardless of such drops in metabolic rate, studies suggest that the activity level of CR animals remain the same or may even increase [31,33,35,40-43]. This is probably because CR animals need to more actively forage when food is scarce [35,44].

Overall, at the beginning of CR, the total energy from food decreases while the mass-specific metabolic energy remains the same, thus, the deposition in mass-specific new biomass (body mass gain, the box with green frame in Figure 1) must be suppressed. As emphasized above, the energy deposited in new biomass is proportional to the energy for synthesizing new biomass. When there is not as much new biomass to synthesize, the organisms do not have to invest much in metabolic biosynthesis. To reiterate: as the metabolic rate and activity levels remain the same, the decreased requirement for syntheses of new biomass allows more energy to be devoted to maintenance. This process, we hypothesize, is the mechanism by which CR animals channel more energy into pathways of maintenance. In other words, the extra energy for maintenance would have otherwise been used to cover the indirect costs of growth (energy for biosynthesis), which is reduced because the direct cost of growth (energy deposited in new biomass) is suppressed by CR. The crucial point is that the metabolic rate remains roughly unchanged or slightly decreased under CR. If metabolic rate is largely reduced (box with red frame), then (1) less energy from food (CR, box with black frame) may not suppress the direct cost of growth (box with green frame), and (2) even if the costs of growth are reduced, extra energy for maintenance may not be guaranteed, since the whole box with red frame is reduced.

Note: all the quantities in Figure 1 are mass-specific. The mass-specific intake rate of food reduces at the initiation of CR, but after a transient period, will approach the same level as in ad libitum fed animals, or even higher [28,43,45-47], which is predicted by the model (see Method and Results sections). Consequently, the effect of CR on channeling additional energy to maintenance also diminishes after the transient period, which is also predicted by our model. To illustrate the energy tradeoffs in Figure 1, for simplicity, we assume that mass-specific metabolic rates are the same in CR and control animals. Again, this assumption is well supported by empirical data [32-34], though, again, there exist some observations indicating that the mass-specific metabolic rate in CR animals decreases slightly [27,30]. In those cases, the decrease in mass-specific metabolic rate is smaller than the decreases in mass-specific biosynthesis cost, thus, the mass-specific maintenance efforts still increase (see Method and Results sections).

In this paper, we address four longstanding questions regarding CR's effects on animals' health maintenance. (1) How does body temperature reduction in CR animals influence CR's effect? (2) What is the relationship between intensity of CR and its effect? (3) Under the same intensity and duration of CR, how is an organism's adult body size correlated to CR's effect? (4) How does the age at which CR begins and the duration of CR influence CR's effect?

## Method

### Metabolic rate and body temperature

Over ontogeny, the resting metabolic rate, *B*_rest_, scales with body mass, *m*, as *B*_rest _= *B*_0_*m*^3/4^, where *B*_0 _is a normalization constant for a given taxon. This scaling relationship is predicted from allometric theories, and supported by data on a diverse set of organisms, including mammals, birds, fish, and mollusks [23,24,48-51]. The normalization coefficient, *B*_0_, exponentially increases with body temperature *B*_0 _~ *e*^-*E*_0_/*KT*^, where *E*_0 _is the average activation energy of metabolism (c. ~0.65 eV), *K *is Boltzmann's constant (8.62 × 10^-5 ^eV/Kelvin), and *T *is body temperature [49,52]. Thus, when assuming equal body temperatures, CR and ad libitum fed animals will have an equal mass-corrected metabolic rate, *B*_0 _= *B*/*m*^3/4^. Some empirical studies have reported mass-corrected metabolic rates as *B*/*m*^3/4^, and some studies reported the values of metabolic rates per body mass, i.e., *B*/*m*. As indicated in the Introduction, empirical studies found that mass-corrected metabolic (*B*/*m*^3/4 ^or *B*/*m*) is roughly the same in CR and control animals; or in a few severe cases, it is reduced by up to 15% in the CR animals. The drop of metabolic rate can be attributed to a drop of the normalization coefficient, *B*_0_, which in turn can be attributed to the drop in body temperature in CR animals, i.e., *B*_0,CR _= *B*_0 _× *e*^-*E*_0_/*K*(1/*T*_CR _- 1/*T*)^, where *B*_0, CR _is the normalization coefficient for CR animals. Using this formalism, we can estimate the body temperature change in response to a change in metabolic rate. For example, in the extreme cases, when mass-corrected metabolic rate, *B*/*m*, decreases by 15%, this equation predicts that the body temperature will decrease by 2~3°*C*. Some empirical studies have reported body temperature drops as slight as ~ 1°*C *in rats [28], 1~1.5°*C*in mice [28,35,53], and 0.5°*C *in Rhesus monkeys [54]. Since most studies show either no temperature drop or a drop of up to 2°*C*in CR animals (see Table 4.21 in [1] Pp 211), we will estimate the CR's effect for 3 cases of body temperature drop, Δ*T *= 0, -1, and -2°*C*.

### CR's effects on growth

Previously, we have developed an energy budget model for understanding how CR retards growth [20]. The outline of this energy budget model is as follows: Based on conservation of energy, West et al [22] proposed that during growth, the whole-organism resting metabolic rate, *B*_rest_, is partitioned between the rate of energy allocated to synthesize new biomass, *B*_syn_, and the rate of energy allocation to maintain existing biomass *B*_maint_, i.e., *B*_rest _= *B*_syn _+ *B*_maint_. The first term can be expressed as *B*_syn _= *E*_m_*dm*/*dt*, where *dm*/*dt *is the growth rate and *E*_m _is the amount of metabolic energy required to synthesize a unit of biomass. The second term can be expressed as *B*_maint _=*B*_m_*m*, where the rate of energy allocated to maintenance, *B*_maint_, is assumed to be linearly proportional to body mass, *m*. Linearity is assumed because the total number of body cells scales linearly with body mass and because, on average, each cell requires approximately the same energy for maintenance [22]. The growth equation therefore, can be written as,(1)

When growth stops, i.e., *dm*/*dt *= O, and an organism reaches its adult mass, *M*, Eq. 1 gives, *B*_0_*M*^3/4 ^=*B*_m_*M*, and *B*_m _= *B*_O_*M*^-1/4^. Solving Eq. 1 yields the growth curve, , where *m*_0 _is the initial body mass at birth. Differences among species are reflected by the different values of *B*_0_, *m*_0_, *M*, and *E*_m_, thus, we see a difference in growth curves.

Hou et al extended the model [21] that partitions the energy assimilated from food between resting metabolic energy and normal activity, and combustion energy stored in the new biomass,(2)

where *A *is the rate of food intake, *B*_tot _is the total metabolic rate, *S *is the rate of energy stored as new biomass, and *E*_c_, different than *E*_m_, is the combustion energy content of a unit biomass (c. ~7000 J/gram). Total metabolic rates can be expressed as *B*_tot _= *B*_rest _+ *B*_act _= *fB*_rest_, where *B*_act _is the rate of energy expenditure for locomotion, feeding, and other activities, and *f*, a dimensionless parameter usually ranging from 2 to 3, reflects the organisms' activity levels.

Combining Eqs. 1 and 2, the food intake rate, *A*, can be expressed as a function of body mass during growth as,(3)

Predictions of food intake as reflected by Eq. 3 are supported by data for mammals and birds of diverse body sizes and taxa [21].

Building on Eqs. 1-3, we modeled how CR retards growth [20]. If CR is intiated at age *τ*, and the amount of metabolic energy intake from food is lowered to a fraction, *β*, of what ad libitum (AL) fed animals consume (*β *ranges from 50%~80% usually), then the food intake rate becomes *A*_CR_(*t*_>τ_) = *β *× *A*(*t*_>τ_), and Eq. 2 becomes,(4)

where *m*_CR_(*t*) is the body mass of CR animals during growth, and the normalization constant *B*_0, CR_, may differ from *B*_0 _due to the body temperature change under CR. Before CR starts, i.e., *t *<*τ*, the growth curves, *m*(*t*) and *m*_CR_(*t*), overlap.

Substituting Eq. 3 into Eq. 4 gives,(5)

**Figure 2:**
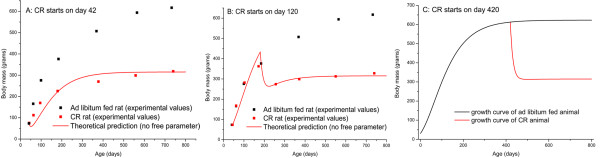
**Growth curves of ad libitum fed and CR rats**. (A) CR starts after d 42; (B) CR starts after d 120; (C) Theoretical prediction of growth curve when CR starts in adulthood (data on ad libitum fed animals from Table 1).

Each organism has four specific characteristics to describe its growth: the metabolic normalization constant, *B*_0_; initial mass, *m*_0_; adult mass, *M*; and energy required to synthesize biomass, *E*_m_. Once these values are obtained from the growth curve of ad libitum fed organisms, *m*_CR_(*t*) will be uniquely determined by Eq. 5, i.e., no additional free parameters are needed. Predictions resulting from Eq. 5 are well supported by empirical data on CR mammals and birds [20]. Here we show an example of retarded growth in rats (Figure 2). Using the growth curve data on ad libitum fed rats from [45], Eq. 5 predicts two growth curves for the CR counterparts with different CR initiation ages: *τ *= 42 d and 120 d (Figure 2A and 2B). The predictions of the CR growth curves are not obtained from data fitting, but rather from the theoretical predictions derived by Eq. 5, again, with no free parameters. In Figure 2C, we show the theoretical prediction for body mass reduction if CR starts after the adult mass is reached. In this case, Eq. 5 predicts the negative mass increase (negative growth) to keep the energy and mass balance. Further validation of Eq. 5 based on additional empirical data from organisms of different taxa and body masses, is available in [20].

### CR's effects on maintenance

Once the energy budget during growth under CR is known and the growth curve is obtained, we can estimate the excess level of energy channeled to maintenance under CR conditions. First, we assume that the dissipative mechanisms of oxidative metabolism and their subsequent deleterious productions, (e.g., reactive oxygen species), cause various forms of molecular and cellular damage. Since the oxygen consumption rate of an organism is proportional to its metabolic rate, *B*, we assume that the rate of damage, *H *(in units of damaged mass/time), is proportional to *B*, i.e., *H *= *ηB*, where *η *is a constant independent of species in units of mass/energy. Second, organisms have biological pathways of maintenance for their integrities (e.g., damage repair, error-checking and correction, et cetera), which require metabolic energy. The rate of maintenance, *R*, (in units of repaired mass/time), is proportional to the rate of energy allocated to maintenance with a coefficient, *ρ*, again, independent of species (in unit of mass/energy), i.e., *R *= *ρB*_maint_. The coefficients *η *and *ρ *are assumed to be constants for a given taxon.

Here, we introduce a mass-specific relative maintenance, which is defined as rate of maintenance per damage per body mass, i.e., (*R*/*m*)/(*H*/*m*) = *R*/*H*. Since growth is retarded, the energy for synthesizing new biomass, *B*_syn_, is reduced. Recalling that the resting metabolic rate, *B*_rest_, remains constant, excess metabolic energy can then be channeled to the maintenance, *B*_maint _(Eq. 1, *B*_rest _= *B*_syn _+ *B*_maint_). We hypothesize that if additional energy is channeled to maintenance pathways, the organisms will have less net damage per body mass and will be in a relatively healthier state. If this is the case, the mass-specific relative maintenance, *R*/*H*, for CR animals should be larger than that of control animals, i.e., *R*_CR_/*H*_CR _>*R*/*H*.

The calculations for mass-specific rate of maintenance *R*, and rate of damage, *H*, are straightforward for ad libitum fed animals. The mass-specific damage rate is *H*/*m *= *ηB*/*m *= *ηB*_O_*m*^-1/4^, recalling *B *= *B*_O_*m*^-3/4 ^(Eq.1). Since *B*_maint _= *B*_m_*m *and *B*_m _= *B*_0_*M*^-1/4 ^(Eq.1), we have the mass-specific maintenance rate, is *R*/*m *= *ρB*_maint_/*m *= *ρB*_O_*M*^-1/4^. For CR animals, the mass-specific damage rate is . The mass-specific maintenance rate for CR animals is more complex because there is no analytic expression for the maintenance rate. However, the maintenance rate is the difference between the resting metabolic rate, *B*_CR_, and the rate of energy spent on biosynthesis, *E*_m_*dm*_CR_/*dt*. Thus, the maintenance rate for CR animals can be expressed as,

So the mass-specific relative maintenance for ad libitum fed animals is , and for CR animals:

. In the last step, we used the relationship , where *B*_CR _is the metabolic rate of CR animals, and *B*_0,CR _= *B*_0 _× *e*^-*E*_0_/*K*(1/*T*_CR _- 1/*T*^.

The ratio of mass-specific maintenance in CR animals relative to that of control animals is therefore,(6)

Once the normal growth curve, *m*(*t*), is empirically obtained from the ad libitum fed animals, the CR growth curve, *m*_CR_(*t*), can be obtained by solving Eq. 5; and the ratio of mass-specific maintenance rates will be determined by Eq. 6 without any arbitrary free parameters.

Before CR starts, the numerator of Eq. 6 is simply (*B*_0_*m*^3/4 ^- *E*_m_*dm*/*dt*)/*B*_0_*m*^3/4^, which can be reduced to (*m*/*M*)^1/4 ^by virtue of the ontogenetic growth equation (Eq.1), so *r*(*t*) = 1. Immediately after CR starts, the growth of the CR animal is suppressed (Eq. 5, Figure 2 and Ref [20]), so the term *E*_m_*dm*_CR_/*dt *in the numerator of Eq. 6 decreases, leading to a quick increase of *r*(*t*) immediately following CR initiation. This means that mass-specifically CR animals will have more energy for maintenance than do control animals. However, the suppression of growth diminishes gradually during the transient period (Figure 2 and Ref [20]), so *r*(*t*), after reaching the maximum, decreases gradually too. After the transient period, the numerator of Eq. 6 can be expressed as . It was proved that at the same age, *t*, after the transient period, *m*(*t*)/*M *= *m*_CR_(*t*)/*M*_CR _(detailed mathematic proof and supporting empirical data are available in [20]), so that the numerator equals the denominator in Eq. 6, and *r*(*t*) reduces to one.

Once the normal growth curve, *m*(*t*), is empirically obtained from ad libitum fed animals, the CR growth curve, *m*_CR_(*t*), can be obtained by solving Eq. 5, and the ratio of mass-specific maintenance rates will be determined by Eq. 6, without the use of any arbitrary free parameters.

## Results

To validate predictions produced by the model, we used publicly available biological data. The symbols, values and data sources are listed in Table 1.

**Table 1:** Value of parameters to carry out Eqs. 5 and 6

symbol	*M *(gram)	*m*_0 _(gram)	*E*_m _(Joule/gram)	*E*_c _(Joule/gram)	*B*_0 _(Watts/gram^3/4)^
Meaning	Adult mass	Birth mass	Energy to synthesize one unit of biomass	Energy content in one unit of biomass	Normalization coefficient of metabolic rate of mammals

Value	600	30	6000	7000	0.022

Source			[21,24]	[22,24]	[51]

### Mass-specific caloric intake between control and CR animals

**Figure 3:**
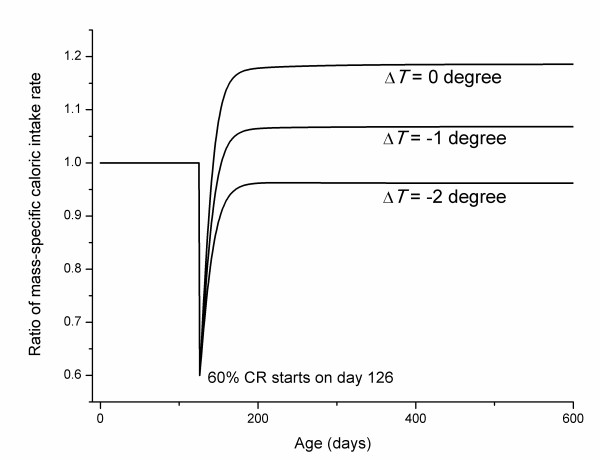
**Ratio of mass-specific caloric intake of CR and ad libitum fed animals for different levels of body temperature drops in CR animals**.

Many studies have shown that CR animals' mass-specific caloric intake is reduced at the initiation of CR, but after a transient period, increases to the approximate level of ad libitum fed counterparts, or in some cases, even higher [28,43,45-47]. Using the growth curves, *m*(*t*) and *m*_CR_(*t*), and food intake rates from Eq. (3), we estimate the ratio of mass-specific intake rates for CR and ad libitum fed animals, and show the effect of body temperature change (Figure 3) (60% CR starts on day 126).

From Figure 3, we see that mass-specific food intake rates for CR animals drop right after CR starts, but show an increase following the transient period. When body temperature does not drop or drops slightly in the CR animals, their mass-specific caloric intake is about 5%-20% higher than ad libitum fed animals, which is in agreement with the empirical observation. When the body temperature drops more than one degree, the mass-specific caloric intake of CR animals is lower than in controls, which is also in agreement with empirical data [28].

### CR channels extra energy to maintenance

In this section, we first estimate the ratio of mass-specific relative maintenance of CR and control animals, *r*, as defined in Eq. 6. This ratio, *r*(*t*), reflects the relative instantaneous rate of net damage in a CR animal compared to that of a control animal. However, damage accumulates over time, so instantaneous rates may not adequately reflect the overall, lifetime effects of CR. To relate this ratio to the effects of CR on lifespan, we integrate *r*(*t*)-1 over time, and assume that the integral, , is positively correlated with the extension of lifespan. Note, if *r*(*t*)-1 = 0, then the extension of lifespan will also be zero. This integral also makes it possible to investigate how restoration of ad libitum feeding after CR would affect lifespan extension. Since restoration of ad libitum feeding often leads to compensatory growth [20], it may have a negative effect on health maintenance, and its counteraction to the overall effects of CR may not be correctly reported by the instantaneous rate. For these reasons, an integral is necessary. Together, we show how drops in body temperature, degree of CR, adult body mass of the species, age of CR initiation, *τ*, and restoration of ad libitum feeding influence the instantaneous relative maintenance ratio as well as the overall effects of CR on maintenance, i.e., *r*(*t*) and .

**Figure 4:**
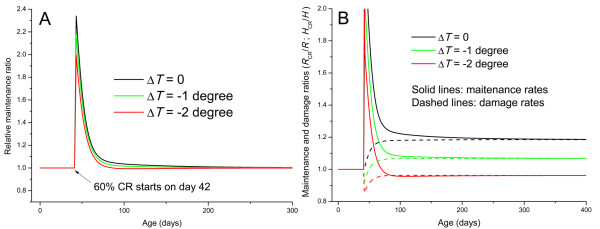
**The influence of body temperature changes on CR's effects**. (A) The relative maintenance ratio, *r*, as a function of age for different body temperature changes. (B) The ratio of maintenance and damage rates as functions of age for different body temperature changes. (In both graphs, the degree of CR, *β *= 0.6, and the CR starting point, τ = 42 d)

In Figure 4A, we plot *r*(*t*) for different levels of body temperature reduction as functions of organisms' age. The ratio of mass-specific relative maintenance, *r*(*t*), increases to its maximum at the initiation of the CR regimen. As organisms get older, this ratio decreases to its original value. During the transition period, CR animals have higher relative maintenance than their ad libitum fed counterparts. This ratio is negatively correlated to body temperature drops. The values of integral, , which are assumed to be positively correlated to lifespan extension, are 18.3, 14.2 and 9.5 for temperature drops of 0°*C*, 1°*C*, and 2°*C*, respectively. This indicates that body temperature drops under CR counteract the effects of lifespan extension. To illustrate the details of temperature influence in Figure 4B we plot the ratio of maintenance rates, *R*_CR_/*R*, and damage rates, *H*_CR_/*H*, for CR and control animals. Here, we assume that temperature will change to a stable level instantaneously as CR begins. As the body temperature of CR animals decreases, the ratio of maintenance rates, *R*_CR_/*R*, also decreases, indicating that lowered body temperatures undermine CR's effects on maintenance. For example, when body temperature drops by 2°*C*, the maintenance ratio decreases to below one (the red solid line), meaning that CR animals allocate less energy to maintenance than do control animals. But, lower body temperature in CR animals also means lower metabolic rate, and thus lower damage rates. So, when body temperature drops by 2°*C*, the damage ratio is also below 1 (as shown by the red dashed line), again, asserting that CR animals have less damage than the control animals. Overall, the relatively lower damage rates and relatively lower maintenance rates in CR animals with low body temperatures lead to the ratio of relative maintenance, *r *(maintenance per damage per body mass), greater or equal to one (Figure 4A).

**Figure 5:**
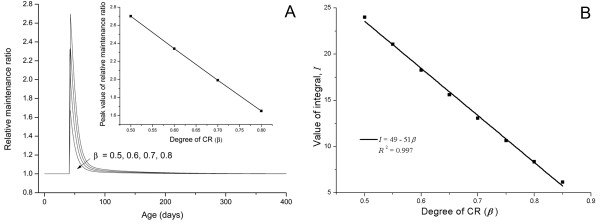
**The effect of CR degree on health maintenance**. (A) The relative maintenance ratio, *r*, for different degrees of CR. The inset shows that the peak value of relative maintenance ratio (right after CR starts) is negatively proportional to the degree of CR. (B)The overall effect, *I*, is inversely proportional to the CR degree. (CR starting point, τ = 42 d, and temperature drop, Δ*T *= 0).

Figure 5A shows the relative maintenance ratio for different degrees of CR, *β*. Results indicate that higher levels of CR (smaller *β*), yield higher relative maintenance ratios, i.e., relatively more energy is allocated to maintenance as CR levels increase. Figure 5B shows that the overall effect of CR on *r*(*t*), , is inversely proportional to the degrees of CR in the range of 0.5 <*β *< 0.8. This range is usually taken in most CR experiments [1-3]. This result is in accord with empirical observations that show an increase in lifespan is positively correlated to the degree of CR (e.g., see [43] and [55]).

**Figure 6:**
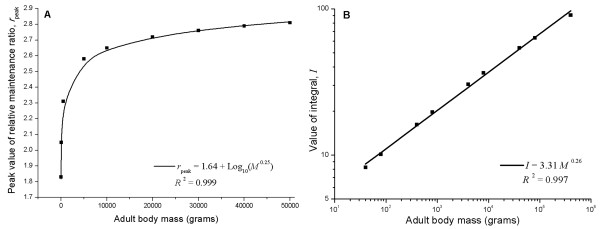
**The effect of adult body mass on health maintenance**. (A) Peak value of relative maintenance ratio is proportional to the logarithm of adult body mass to a 1/4 power. The dots are the calculated values from Eqs. 5 and 6. (B) The overall effect, *I*, scales with adult body mass to a 1/4 power. (The degree of CR, *β *= 0.6, the CR starting point, τ = 42 d, and temperature drop, Δ*T *= 0).

CR has been tested in organisms with a wide spectrum of body masses. Until now, no study had investigated how individual body masses modulate the effects of CR. In Figure 6A, we plot the peak value of relative maintenance ratios versus their adult body mass in ad libitum fed organisms, ranging over 4 orders of magnitude. As shown, the peak value is proportional to the logarithm of the body mass to the ¼ power, log*m*^1/4^. Figure 6B shows that the integral, *I*, scales with the body mass to a power close to 1/4, *I *= 3.31*M*^0.26^, where *M *is in grams. This result suggests that the mass-specific excess available for maintenance increases with body mass, so that CR might potentially increase lifespan more for larger organisms; an observation yet to be corroborated experimentally.

**Figure 7:**
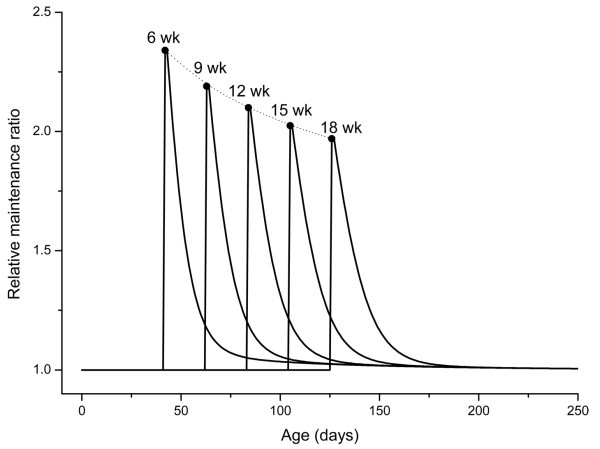
**The relative maintenance ratio for different ages at which CR is initiated (the degree of CR, *β *= 0.6, and temperature drop, Δ*T *= 0)**.

In Figure 7, we investigate the influence of the age at which CR is initiated on the relative maintenance ratio. Curves indicate that CR has a positive effect on maintenance regardless of whether the CR regime starts late in life, but that the effects decrease as the age of initiation gets higher. In other words, the peak value of the relative maintenance rate is negatively correlated to the starting age, which agrees with empirical observations that the benefits of late-life CR, such as lifespan extension or age-related disease retardation, are not as great as in cases of early-life CR initiation [45,56-60].

**Figure 8:**
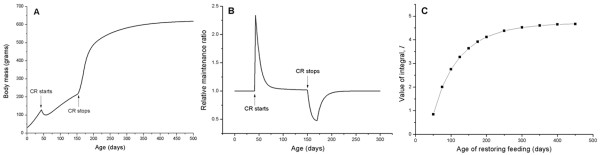
**The effect of restoring ad libitum feeding**. (A) Growth is suppressed after CR is initiated. When ad libitum feeding is restored (CR stops), compensatory growth occurs; (B) The relative maintenance ratio, *r*(*t*), versus age. *r*(*t*) drops immediately after CR stops on day 150, and increases gradually back to one; (C) The overall effect of CR, *I*, at different age of restoring feeding. (The degree of CR, *β *= 0.6, the CR starting point, τ = 42 d, and temperature drop, Δ*T *= 0).

Finally, we study the effects of restoring ad libitum feeding after CR, which starts on day 42. First in Figure 8A we show that restoring ad libitum food supplies on day 150 leads to a compensatory growth, as expected [20,61]. Numerous mammal and clinical studies suggest that compensatory growth often causes poor health maintenance, increases the risk factor for adult diseases and shortens lifespan [e.g., see [62-64]]. The model presented here predicts an energy tradeoff between the compensatory growth and health maintenance. So, in Figure 8B we see that the relative maintenance ratio, *r*(*t*), drops immediately after ad libitum food supplies are restored, and increases gradually back to one. However the drop of *r*(*t*) due to the compensatory growth does not completely counteract the increase of *r*(*t*) due to CR. To quantitatively illustrate the overall effects of CR and restoring feeding, we calculate the integral, , for different age of restoring feeding, *T*, and plot *I *versus *T *in Figure 8C. We see that if restoration of feeding occurs immediately after CR starts, e.g., on day 50, the value of *I *is very small, indicating that the positive effect of CR is almost completely undermined by the negative effect of compensatory growth. When the duration of CR increases, i.e., when restoration of ad libitum feeding occurs later in life, the overall effect, *I*, increases and then reaches a plateau. This is because as age increases, the potential for growth decreases [20,61], in other words, the compensatory growth caused by restoration of ad libitum food supplies later in life does not cost as much energy as that which occurs earlier in life. This result agrees with empirical observations. For example, Yu et al [45] reported that the extension of rats' lifespan by CR that began on day 42 and stopped on day 180 was 15%, whereas lifelong-CR extended lifespan by 50%.

## Discussion

Many researchers have proposed theories of aging and CR's effects on retarding aging from an energetic viewpoint. The earliest theory is probably the Rate of Living Theory [65-67], which assumes a fixed amount of metabolic potential for every living organism, so that the higher the mass-specific metabolic rate, the faster the rate of aging and the shorter the lifespan. This theory is largely supported by extensive field data from invertebrates, fish, birds and mammals [51,68,69], especially when comparisons are across species [70]. However, it fails to explain the effects of CR on lifespan extension, because it has been shown that there is very little, if any, reduction in mass-specific metabolic rate in CR animals, and the degree of the reduction is not sufficient to explain the significant increase in lifespan.

Models based on the Disposable Soma Hypothesis suggest that CR's effects are not reaped by reducing mass-specific metabolic rate, but by inducing energy tradeoffs between maintenance and other life history traits. Those models can qualitatively explain CR's effects, but have some limitations. For example, one of the best-known energy tradeoff models by Shanley and Kirkwood (S-K model; [11]) assumes that CR suppresses the effort of reproduction and channels extra energy to maintenance of soma. While the model explains the CR effects on female rats, it fails to account for the observations of CR's effects on male animals, whose reproductive efforts are not influenced by CR as much as the females [19,71-73]. The model also crucially depends on a mathematical relationship between food availability and the probability of infant survival, which may be difficult to justify [19]. Moreover, the S-K model and other energy tradeoff models [10-14] all contain a few free-adjusting parameters, which either have no biological meaning or are difficult to link to real biological data.

The model presented in this paper, in contrast, uses parameters that are derived from fundamental physiological properties and can be quantitatively obtained from metabolic measurements. The alteration of energy allocation during CR in this model is not pre-assumed, but predicted by the growth and food uptake and partition equations. Moreover, the model makes several testable predictions. Most predictions derived from this model are in agreement with empirical observations (Figure 2, 3, 5, 7, and 8), and others, where no data is available yet, (Figure 4 and 6) can be used to guide future research. The results in Figure 4 indicate a positive correlation between lifespan or health maintenance and body temperature under CR conditions, which seems to contradict the common belief that lower body temperature extends lifespan [for example, see [74]]. However, the negative correlation between body temperature and lifespan has only been observed in animals fed ad libitum. No experiments have been done to investigate lifespan of endotherms with different body temperatures under CR conditions. CR extends lifespan and in many cases lowers body temperature, but as far as we are concerned there is no evidence showing lowered body temperature as the mechanism underlying CR's effect on health maintenance. The different correlation between body temperature and lifespan under CR and AL is probably due to the different role of body temperature in energy budgeting during ontogeny. Under ad libitum conditions, metabolic rate is the dominant constraint on growth (Eq. 1), so body temperature, via metabolic rate, is positively correlated to the growth rate. Under CR, however, food intake has more influence on growth. Due to the tradeoff between metabolism and new biomass storage, higher body temperature and thus higher metabolism, leads to slower growth (Figure 1 and Eq. 4). This negative correlation between metabolism and growth has been reported in experiments on rats, in which food was restricted and experimentally elevated metabolic rates were found to be associated with severely reduced growth [75]. Hence, based on our model, the retardation of growth channels extra energy to maintenance.

In our model, animals' activity level under CR is assumed to be unchanged, which is supported by most empirical studies (see review in [20]). Nonetheless, Figure 1 provides a qualitative description of what might happen in the exceptional case that activity level does change. First, the resting metabolic rate, which is sum of biosynthesis and maintenance in Figure 1, is determined by body mass and body temperature and therefore will not change if the activity level changes. Therefore, when a limited food supply yields CR conditions, the increased activity level would suppress growth (deposition in new biomass) to a relatively greater extent. In turn, more energy will be channeled from biosynthesis to maintenance. On the other hand, in the case that activity levels are decreased, there will be more room for deposition in new biomass, and animals will allocate more energy to biosynthesis. With the fixed resting metabolism, more energy allocated to biosynthesis means less energy is allocated to maintenance.

This model is built on the previous energy partition model, Eq.3 [21], and model of growth under CR, Eq. 5 [20], both of which are validated for mammals and birds, but yet to be tested using data for invertebrates. Studies on *Drosophila *by Mair et al [76] and Lee et al [77] have shown that the lifespan extension by reduction of particular nutrition in food, such as protein or sugar, is not attributed to the reduction of total caloric intake. O'Brien et al [78] have tested the disposable soma hypothesis on *Drosophila*, and found that while flies under dietary restriction invested less to reproduction, they also invested less to soma maintenance compared to their ad libitum fed counterparts. These aging studies on *Drosophila *suggested that the alteration of energy budget in invertebrates under dietary restriction may be inconsistent with the present model. So, our model will need further modification and extra empirical-grounded assumptions before it can be applied to invertebrates.

We need to emphasize that our model is fundamentally different than the "growth retardation hypothesis" [61], which proposes that CR extends lifespan simply by slowing growth and extending the growth period. First, the growth retardation hypothesis is based on the statistic and descriptive correlations between temporal lengths of growth and lifespan, whereas this model is based on the fundamental mechanisms of energy tradeoffs between growth and maintenance. Second, the growth retardation hypothesis cannot explain the observation that CR after maturity or in midlife; at a time when it will not affect growth or interfere with development, also extends life. On the other hand, this model predicts that late-CR is also beneficial, and its effect is not as marked as when CR starts soon after birth (Figure 7).

One important feature of CR revealed by our model is that CR's effects are mounted rapidly and then diminished gradually, which can be seen from Figure 4, 5, and 7. This agrees with empirical observations. For example, one result of CR is hypercorticism, shown to slow aging and carcinogenesis by reducing rates of intracellular glycoxidation and oxidative damage, as well as through its anti-mitotic and anti-inflammatory functions [47]. These effects only occur during the early stages of CR [79,80]. Also, the effects of CR on biomarkers of mitogenesis are generally consistent with the occurrence of hypercorticism during early but not late stages of CR (see review in [47]). The rational for the decrease of the relative maintenance ratio is that after a transient period, the mass-specific food intake rate is roughly the same in CR and ad libitum fed animals, as shown in Figure 2 and empirical data, thus, the CR effects on suppressing growth and channeling extra energy to maintenance of soma diminishes. However, the fact that the relative maintenance ratio, *r*, decreases to 1, does not mean that CR animals' health status will be the same as the control animal after the transient period. Health status and lifespan are determined by the battle between damage and repair, which is an accumulative process. As we showed in Figure 5, 6 and 8, the overall, lifetime effects of CR on health maintenance, namely, the integral  is greater than 1. So, the high relative maintenance ratio during early phase of CR and high overall lifetime integral, *I*, result in less net damage, which maintains a lower level late in life and leads to better health and longer lifespan.

There exist several proximate hypotheses for CR's effects at the cellular level, including the oxidative damage attenuation hypothesis [81-83], altered glucose-insulin system hypothesis [84-87], alteration of the growth hormone-IGF-1 axis hypothesis [88-90], et cetera. These specific hypotheses, which address specific damaging or defending processes underlying aging, have recently been synthesized into the general hormesis hypothesis [3,4,91,92]. Hormesis refers to the phenomenon whereby a usually detrimental environmental agent changes its role to provide beneficial effects when administered at low intensities or concentrations [93]. CR, as a low-intensity stressor, activates hormetic mechanisms in organisms, defending them against a variety of adversities and, in the case of long-term exposure, retarding aging [3]. While this hypothesis has gained some empirical support, it remains somewhat descriptive. Our model makes a significant contribution to the general hormesis hypothesis by illustrating the underlying mechanism from an energetic viewpoint.

## Conclusion

Our model reveals how an animal alters its energy budget when food availability is low, and offers better understanding of the tradeoffs between growth and somatic maintenance; therefore shedding new light on aging research from an energetic viewpoint.

## Abbreviations

CR: caloric restriction; AL: ad libitum; S-K model: Shanley and Kirkwood model

## Authors' contributions

CH contributed to the conception, designed the research, carried out the calculation, analyzed data and wrote the paper; KB analyzed data and wrote the paper; AB carried out the calculation and wrote the paper. All authors read and approved the final manuscript.
